# How can technology leverage university teaching & learning innovation? A longitudinal case study of diffusion of technology innovation from the knowledge creation perspective

**DOI:** 10.1007/s10639-023-11780-y

**Published:** 2023-04-29

**Authors:** Xiaolei Zhang, Shuangye Chen, Xiaoxiao Wang

**Affiliations:** 1 School of Teacher Education, Faculty of Education, East China Normal University, Room 922, Wenke Building, Putuo District, Shanghai, 200026 China; 2 Institute of Curriculum and Instruction, East China Normal University, Room 1603, Wenke Building, Putuo District, Shanghai, 200062 China; 3 Online Education Center, Tsinghua University, Haidian District, Beijing, 100084 China

**Keywords:** Institutional innovation, Knowledge creation, Diffusion of innovation, Technology-enhanced teaching & learning, Middle leadership, Higher education, Longitudinal case study

## Abstract

This paper expands the innovation diffusion framework by adding a conceptual and empirical exploration of knowledge creation into understanding university technology-enhanced teaching and learning innovation. Institutional innovation research has largely focused on people and products while neglecting the underlying knowledge creation process for innovation that substantiates and sustains the diffusion of innovation across stages. Guided by a combined framework of organisational knowledge creation theory with the diffusion of technology-enhanced teaching & learning (T&L) innovation, this 4-year longitudinal qualitative study focused on a Chinese case of Tsinghua University, which has pioneered the adoption of digital teaching and learning, and generating exemplary sustainable whole-institutional teaching and learning innovation. We explored how technology leverages the interactions between technologies, adopters, and leadership within a university to build capacities for digital T&L innovation by tracing the technology innovation trajectory of Tsinghua University. The case study identified four stages of knowledge creation related to technology adoption and innovation. Of these stages, knowledge externalisation processes were found to be critical for leveraging the co-creation of knowledge for institutional innovation in the university context. Additionally, the study showed that the middle-up-down leadership strategy and middle managements’ knowledge management ability facilitated the sustainable transition from individual and group exploration to organisational innovation. The implications for strategic technology adoption and sustainable teaching and learning innovation in the university contexts are also discussed.

## Introduction

Over the last decade, digital technologies, ranging from massive open online courses (MOOCs) and blended instruction practice (BIP) to learning analytics (LA), have shaped how teaching and learning are designed, supported, and achieved in higher education institutions (HEIs). However, despite sufficient funding and strong external drivers (such as the COVID-19 pandemic), technology adoption initiatives in many universities seem to have been implemented as temporary reactive projects or bricolage (Louvel, 2013), rather than as part of proactive or transformative strategies for institutional innovation (Schophuizen et al., 2018; Watermeyer et al., 2021).

Previous studies of digital technology adoption in universities have focused on either the individual factors that affect facultys adoption of technologies (Ashrafzadeh & Sayadian, 2015; Kopcha et al., 2016; Ocak, 2011) or the implementation of institutional visions and strategies for technology-enhanced teaching and learning (TETL) (Graham et al., 2013; Porter et al., 2016). Relatively few studies have examined the dynamic underlying organisational process responses to the advent of online tools for teaching and learning in HEIs (Liu et al., 2020).

Instead of focusing on an either-or strategy, this study foregrounds TETL innovation as an organisational learning and knowledge creation process. In this dynamic process, technology, as an outside-in factor, may leverage people within HEIs to learn, interact, overcome challenges, and build the capacity to fulfil digital teaching and learning (T&L) innovations from the inside out. Linking organisational knowledge creation theory (Nonaka et al., 2001) with the diffusion of the TETL innovation framework in HEIs (Graham et al., 2013; Rogers, 2003), we attempt to disentangle how the interactions between people, tools, and leadership produce shared knowledge of not only technological tools but also new pedagogical practices based on these tools that enable sustainable institutional teaching innovation.

In line with the aim of uncovering processes and mechanisms, we adopted a longitudinal case study method for an in-depth exploration of technology adoption at Tsinghua University, a pioneering university in China in initiating and sustaining institutional innovation in digital teaching and learning. Over the last decade, the Ministry of Education in China has committed to the large-scaled digitalization of teaching and learning in higher education. Tsinghua University launched its MOOC platform in the early 2010s, underwent an exploratory online education process, and moved to a whole-institution technology-supported teaching innovation during the COVID-19 pandemic.

This study was motivated by observations of the university pioneering national TETL innovation and leading inside-out technology innovation in response to the pandemic after a prolonged period of technology adoption. We thus propose the following research question:

- *How did the technology adoption become the inside-out institutional teaching innovation in a university?*

The dynamic processes of knowledge creation and capacity building through technology adoption and innovation to achieve a sustainable digital transformation in T&L were carefully examined.

## Literature review: Technology adoption for institutional teaching innovation in HEIs

Previous studies have indicated that innovation adoption models often focus on individual faculty members (especially tinkerers and champions who have been pioneers or central to this journey) and the factors shaping their adoption (Ashrafzadeh & Sayadian, 2015; Fraser, 2019; Kopcha et al., 2016). Others have focused on institutions’ implementation, showing that universities can accumulate multifaceted resources and experience through the process of technology adoption (e.g., infrastructure, availability of data, institutional culture, and buy-in from stakeholders) (Herodotou et al., 2020; Porter et al., 2016), while overcoming a diverse set of challenges, including resistance to change (Salmon, 2005) and a lack of human capacity to engage with technology-supported T&L (Aitchison et al., 2020; Schophuizen et al., 2018), when adopting technology. Nevertheless, such a disconnection between top-down implementation, which often has limited staff uptake, and bottom-up exploration, which often faces difficulties in communicating and scaling up innovations, can inhibit the growth of technological innovation, transitioning from individual exploration to institutionalization.

Scholars have recently refined this *either-or* typology, arguing that the adoption of digital technology within HEIs is a complex process influenced by learning technologies, faculties, leadership, contexts, and institutional strategies (Tsai et al., 2021; Vigentini et al., 2020). More integrative perspectives have been adopted regarding approaches to technology initiatives in HELs, activity theory (Yamagata-Lynch et al., 2015), the SHEILA framework (Vigentini et al., 2020), and complex leadership theories (Tsai et al., 2019). However, few of them regard TETL innovation as an organisational learning and strategic change process (Bøe et al., 2021; Liu et al., 2020) and demonstrated how the fluid interactions between technologies, people, and leadership in the technology innovation of T & L drive the institutional change happen within university contexts (Aitchison et al., 2020). That is to say, to understand the trajectory and mechanism of strategically initiating and sustaining institutional technology-supported teaching innovation, there remains a need for research using sophisticated analytical frameworks to uncover the ‘black box’ underlying the dynamic interactions between individuals and organisations in relation to technology adoption, which creates innovation in HEIs. 

## Theoretical framework: Organisational knowledge creation and institutional technology adoption for innovation

To reveal how the dynamic interactions between individuals and organisations regarding technology adoption evolve and produce innovation, we combine Nonaka et al. (2001), Nonaka et al. (2006)) organisational knowledge creation theory with the diffusion of TETL innovation framework in HEIs (Frei-Landau et al., 2022; Graham et al., 2013; Pinho et al., 2021; Rogers, 2003) to conceptualise the dynamics and innovation in universities.

According to organisational knowledge creation theory, organisational knowledge matters in organisational innovation; an organisation creates knowledge through the interactions between explicit and tacit knowledge across different levels of knowledge-creating entities (individual, group, organisational) (Songkram & Chootongchai, 2020). The interaction between the two types of knowledge (explicit and tacit) (Polanyi, 1966) is known as knowledge conversion. There are four modes of knowledge conversion for innovation: socialisation, externalisation, combination, and internalisation (SECI) (Songkram & Chootongchai, 2020). *Socialisation* happens when individuals share feelings, experiences, and/or physical knowledge, thereby creating shared tacit knowledge. Experiential knowledge is built through the process of socialisation. *Externalisation* occurs when individuals articulate tacit knowledge as explicit concepts at the group level through dialogue, reflection, deductive thinking, and the use of metaphors, models, or approaches, thereby engaging in concept creation. Conceptual knowledge is built through the process of externalisation. *Combination* is the process of converting discrete elements of explicit knowledge into more complex bodies of explicit knowledge through collective and virtual interactions. Systematic knowledge is made explicit in documents and databases through the process of combination, which helps to make knowledge transferable. *Internalisation* is the process of embodying explicit knowledge, which exists at the organisation level, into tacit knowledge, which exists at the individual level, through personal interactions and learning-by-doing. Operational knowledge is created and shared through internalisation. According to Nonaka et al. (2001), Nonaka et al. (2006)), through these conversion processes, tacit and explicit knowledge expand in both quality and quantity in contexts where interactions between individuals and their environments occur sustainably.

Linking the four modes of knowledge conversion with different stages of diffusion of TETL innovation (Graham et al., 2013; Rogers, 2003) shows that various levels of institutions move from interest in TETL towards mature institutionalization (Table 1).

**Table 1:** Guiding framework linking the knowledge conversions with stages of diffusion of TETL innovation

Unit of analysis	Types of knowledge	Knowledge conversion	Stage 1 Awareness of the new idea through socialisation	Stage 2 Early exploration of the novel technology through externalisation	Stage 3 Mature innovation through combination & internalisation
Individual	Personal/operational knowledge Experiential knowledge	Internalisation Socialisation	➓ Primary focus on traditional classroom and course development ➓ Individual faculty/department chairs informally identify, concern and discuss TETL	➓ Increased focus on TETL for faculty and students ➓ Individual faculties do experiments and build courses with new policies and practices	➓ Wide-spread faculty adoption & innovation
Group/team	Conceptual knowledge	Externalisation	➓ No uniform definition and policy of TETL in place	➓ Administrators identify the purpose to motivate institutional innovation, restructure appropriate structures to support the innovation	➓ Administrative refinement of purpose for continued promotion of TETL ➓ Robust policy, structures, evaluations for TETL innovation
Organization	System knowledge	Combination	➓ No institutional model established	➓ Limited institutional regulation and evaluation addressing the TETL innovation	➓ Well-established technological support, structure, and resources for routinising the TETL innovation

Authors’ own based on Nonaka et al. (2006), Graham et al. (2013) and Rogers (2003)

### Stage 1 Awareness of the new idea through socialisation

At this stage, an institution may be aware of the challenges, consider the TETL as an innovative solution, and provide limited support for individual faculty exploring the new practices, but it has no institutional strategy regarding it. *Socialisation* occurs when department chairs and faculty members share concerns about the effects of online courses on campus teaching practices and informally discuss new ways to develop TETL programs (Nonaka et al., 2001; Rogers, 2003).

### Stage 2 Early exploration of the novel technology through externalisation

At this stage, individual faculty members do experiments with new policies and practices, while university administrators identify the purpose of motivating institutional innovation and try to restructure appropriate governance structures to support the TETL initiatives (Graham et al., 2013; Rogers, 2003). When administrators, IT staff, faculty members, and HEIs students become involved in communities that discuss innovative technology-supported programs and form a consensus on their use, *externalisation* processes occurs (Herodotou et al., 2020; Nonaka et al., 2001; Vigentini et al., 2020).

### Stage 3 Mature innovation through combination & internalisation

At this stage, a *combination* process occurs when well-established TETL strategies, structure, and support are integral to university operations; when faculty and other stakeholders integrate online course designs, and when task forces create learning analytics tools (LA) and infrastructures to support online program development or produce conceptual artefacts for advancing T & L (i.e., product plans, database, manuals) (Nonaka et al., 2001; Paavola et al., 2004). Reforming university rules and improving the institutional culture for technological innovation promotes *internalisation* (Nonaka et al., 2001; Porter et al., 2016; Yamagata-Lynch et al., 2015). Rogers (2003) describes as routinising in this process model for organisational innovation.

In this study, we seeks to identify empirically how technology adoption happens and becomes an inside-out institutional innovation based on an analytical framework (Table 1). Through a longitudinal case study, we investigate how a successful instance of a university experiencing the dynamic processes of knowledge conversion and creation in relation to technology adoption and innovation. Specifically, how dynamic interactions between individuals and organisations in relation to technology adoption promote the knowledge creation spiral that facilitates institutional teaching innovation.

## Methods and data

### Research design and approach

This qualitative 4-year longitudinal case study (2017–2020) attempts to examine the university’s nearly 7-year-long technology adoption initiative from 2013 to 2020. Based on fieldwork, the research design for this study included three parts: (a) collecting archival data (2013–2020) ranging from MOOC and BIP to hybrid online T &L initiatives of the case university; (b) interviewing key administrators, active faculty members, stakeholders, and student tutors about their experiences (and/or recollecting their experiences) with online education initiatives (2017–2020); and (c) participant observation of relevant both online and offline meetings, classes, and training sessions over the research period.

Of the three authors, one played a critical role in the initiative as both researcher and practitioner. The other two acted as researchers and participant observers. We frequently reflected on the ethical dilemmas involved in simultaneously being a researcher, participant observer, and practitioner (Labaree, 2002). The multiple roles generated insider and outsider tensions in analyzing cases that was still unfolding in unpredictable development. Therefore, we frequently engaged in reflective discussions throughout the data collection in the field and data analysis processes.

### Case selection and data

Tsinghua University is one of the top research universities in China. It launched China’s first open online education initiative in 2013, followed by a university-supported MOOC platform, XMOOC. This study started in 2017, when Tsinghua University accelerated its experimentation with technology-supported T&L in response to ambitious national policies aimed at stimulating online education in Chinese HEIs.

We chose Tsinghua University for our case study because it has pioneered educational technology innovations in China. Between 2013 and 2020, Tsinghua University was the leader in technology-supported T&L, including developing MOOC platforms, integrating MOOCs with BIP, creating an LA system (RClass) to support BIP, and creating a hybrid model of online education. These extensive digital T&L initiatives allowed it to firstly implement a whole-institution digital educational transformation after the Covid-19 crisis. Its rich experiences and knowledge products made Tsinghua University a national driver of technological entrepreneurship, leading other HEIs to move T & L online and join in the technological innovation after Covid-19 (Coates et al., 2022).

Data collection for this longitudinal case study was divided into four phases: November-December 2017; February-March 2018; April-July 2019; and February-April, August-September, and December 2020. Table 2 provides detailed information on the data collection process.

**Table 2:** Information of interviewees and interviews

Faculty	Gender	Teaching/working experience (year)	Discipline	Position	Technology adoption and innovation (Year)			Online teaching committee member	Records of interview meetings
					MOOC initiatives	BIP initiatives	Hybrid online T&L initiatives		
Prof. Heng	Male	> 24	Computer science	Professor	2013	2015	2020	No	Nov, 2017
Prof. Xu	Male	16–23	Electrical engineering	Professor	2013	2015	2020	Yes	June, 2019 April, 2020
Prof. Fan	Female	> 24	Language art	Associate Professor	2013	2015	2020	Yes	Nov, 2017
Prof. Yu	Female	8–15	Medical science	Associate Professor	2014	2016	2020	No	Nov, 2017
Prof. Jiang	Male	8–15	Communication studies	Associate Professor	2015	2016	2020	No	Feb, 2018
Prof. Geng	Male	8–15	History	Associate Professor	2015	2016	2020	No	Nov, 2017
Prof. Nan	Male	8–15	Political science	Associate Professor	N/A	2017	2020	Yes	March, 2018 April, 2020
A-Lan	Female	8–15	Higher education administration	Director of Digital Learning Office	2013	2015	2020	No	Dec, 2017 March, 2018 June, 2019 April, 2020 Dec, 2020
A-Nong	Male	0–3	Higher education administration	Secretary of the Director of the Digital Learning Office	N/A	N/A	2020	No	Feb, 2018 June, 2019 April, 2020
ET-Xue	Male	4–7	Computer science	Technological assistant	N/A	2015	2020	No	March, 2018
ST-Sang	Male	0–3	Political science	Graduate student	2014	2015	N/A	No	Nov, 2017
ST-Tu	Female	0–3	Educational studies	Graduate student	N/A	2017	2020	No	Nov, 2017 July, 2019 April, 2020
ST-Lee	Female	0–3	Biology	Graduate student	N/A	N/A	2020	No	June, 2019 Dec, 2020
ST-Hu	Male	0–3	Electrical engineering	Graduate student	N/A	N/A	2020	No	Dec, 2020

The semi-structured interviews focused on (1) motivations, chronological processes, and knowledge development related to the university’s technology innovations; and (2) managerial approaches to T&L technology innovations. We conducted 25 interviews with 14 individuals; 7 faculty members (from the fields of computer science, electrical engineering, medical science, political economics, language studies, and communication studies), 2 administrators in the Digital Learning Office, 4 student tutors, and 1 education technology specialist at Tsinghua University. All interviews were conducted in 2017, 2018, 2019, and 2020. Of these interviewees, 2 faculty members, 2 administrators, and 2 student tutors were interviewed three to five times over the course of the study, as almost all were engaged in the initiative before 2015 and continued to play key roles in 2020. Other informants were interviewed once during the 4 years (Table 3). In addition, we conducted random interviews during the participating observations which were not included in the formal semi-structured interviews and recorded as observational data. All participants orally approved the interviews. The names of the participants were anonymised. Each semi-structured interview lasted 35-90 minutes (face-to-face and online). To protect our informants and ensure confidentiality, pseudonyms are used for all informants.

**Table 3:** Description of data sources

Data source	Description
In-depth interviews	25 interviews were conducted in 2017 (7 interviews), 2018 (5 interviews), 2019 (5 interviews), and 2020 (8 interviews) The following interview questions were asked 1. Institutional level questions (The objective was to gain an inter-subjective understanding of the nature and the process of the technology-based initiatives in your university from the people who were actually involved in the initiatives.) ➓ When, why, and how did your university embark on technology-based initiatives? ➓ When, why, and how did you become involved in technology-based initiatives at your university? ➓ Has there been any change in your understanding of technology-based teaching and learning innovations at your university as a result of the initiative? 2. Individual level questions (The purpose was to understand how learning-by-doing moved forward technology-based innovation in and across the university.) ➓ What managerial/individual/institutional/technological/communal factors influenced your involvement in technology-based initiatives at your university? How? ➓ What did you learn from the initiatives? ➓ What has your university learned and gained from the initiatives?
Participant observations	Participating in and observing 18 activities in both online and offline approaches such as the following 1. Lunch seminars, workshops, and forums where participants in lively discussions contextualised the challenges that may influence innovations. (2017–2020/4) 2. Lesson observations and after-class discussions that involved close interaction amongst the members in concretising artefacts (e.g., ‘RClass’). (2017–2018/2; 2020/2–9) 3. R&D task force which conducted evidence-based research projects (e.g., innovative blended learning projects, student self-regulated learning under the technology-enhanced learning environments, etc.) about technology-based teaching and learning in and across the university. (2018–2020/12)
Participation	Student tutor training programs (2018–2020). The goal was to experience how the members interacted and were immersed in the activities
Archive/Document Review	1. Obtained official publications that documented the innovative practices at the case university, including speeches of university leaders. (2013–2020) 2. Reviewed the university websites, WeChat public board accounts, and press releases (e.g., electronic newsletters) about MOOCs at the case university, the XMOOC Platform, and RClass platforms. (2013–2020)

Observational data were collected from 2017 to 2020 through participation in lunch seminars (4), (online) lesson observations (2), and (online) conferences (12) on online education at Tsinghua University, with administrators, teachers, students, and experts. We also informally talked to people during the participating observations (e.g. we discussed the T&L with teachers, students and tutors after lesson observations). All observations and causal talks were recorded in the form of observation notes and memos, which were compiled, coded, and analysed.

We also collected and analysed official documents and archival data such as institutional policies, annual reports, online education newsletters, and related records in the official WeChat public account of Tsinghua University and the XMOOC Platform from 2013 to 2020. Speeches were collected from university leaders to explore their perceptions of and strategies for technology initiatives. 

### Data analysis

Data analysis was performed continuously throughout the data collection phase. The coding process involved three steps: open coding, axial coding, and selective coding (Strauss & Corbin, 1998). This involved reading and rereading the textual data numerous times to identify initial codes and themes. During the early stages of the analysis, 179 open codes were developed. These codes were grouped into 54 initial sub-themes in the axial coding step, based on similarities in their descriptions. We then applied knowledge-creation theory (Nonaka et al., 2001) and the diffusion of TETL innovation framework (Graham et al., 2013; Rogers, 2003) to our data, unpacked overlapping sub-themes as 41 sub-themes, and clustered the analytical sub-themes into four major themes (shown in Table 3): “top-down awareness through combination and internalisation (2013–2014),” “bottom-up exploration through internalisation and socialisation (2014–2016),” “growing middle-up-down approach for continuous adoption through externalisation (2016–2019),” and “strong middle-up-down approach for mature innovation through combination and internalisation (after 2020).” The major themes and supporting sub-themes are presented as headings in the Findings section. As noted, informed by the theoretical framework, we used an analysis-based rationale to code and analyse the data. The four defined stages mainly depended on sub-themes rather than data collection timelines, although the stages also had timelines.

Throughout our data collection and analysis, we ensured trustworthiness by extending our engagement in the field and collecting multiple types of data to triangulate the evidence through the involvement of more than one investigator. We did not attempt to generalise our findings or draw conclusions regarding the best model for technology initiatives in HEIs. We also do not claim that the perspectives presented in this article are the only possible interpretations of the observed phenomena.

## Findings

In this section, we present the four stages of knowledge conversion and the creation process across the organisation, group and individual levels. We also identify the directions of the top-down, bottom-up, and middle-up-down knowledge process in each stage. The findings summarised in Table 4 are grounded in the collected data and guided by the framework synthesised in Table 1.

**Table 4:**
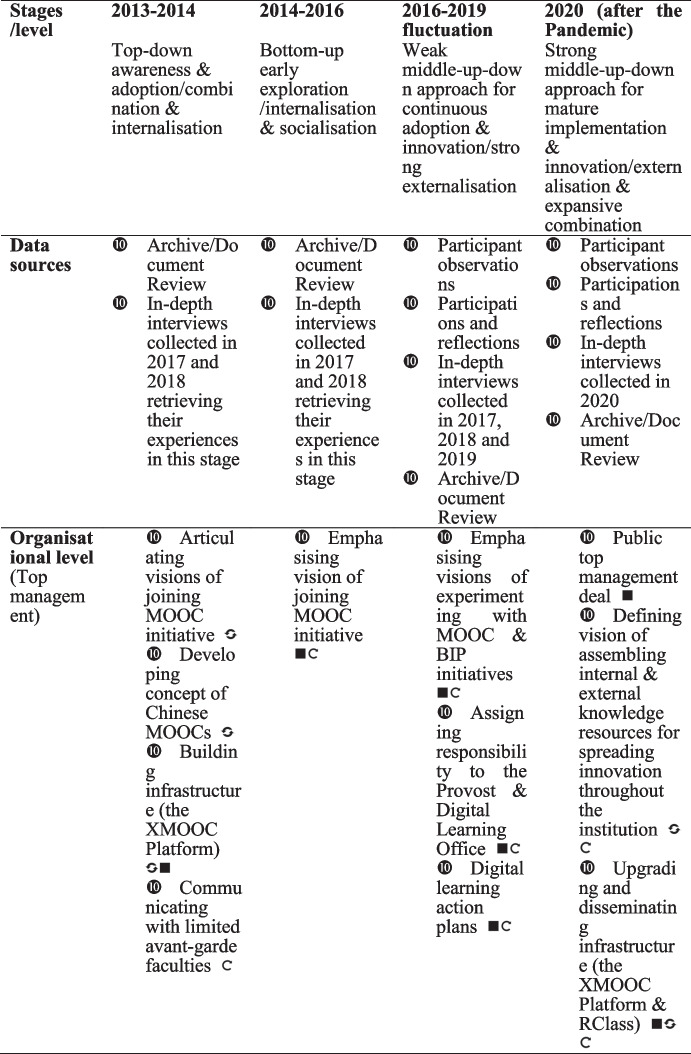

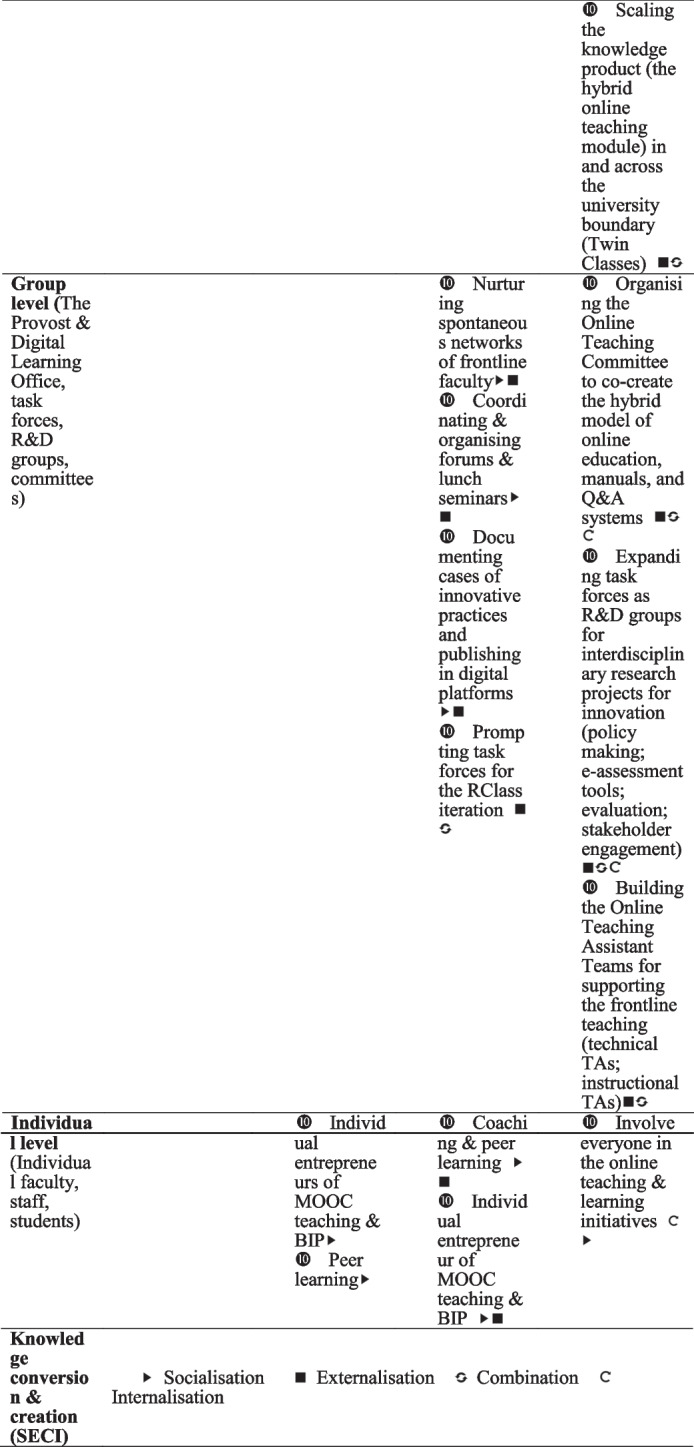
Four stages of knowledge conversion & creation processes for online technology innovation at Tsinghua University

### Stage 1: Top-down awareness and adoption through combination and internalisation (2013–2014)

#### Proposing digital T&L visions and articulating the Outside-In MOOC concept

In 2012, “the year of the MOOC,” prestigious international universities were partnering with MOOC platforms (e.g., Coursera) and online learning was in the process of being scaled up (Pappano, 2012). By that time, the then-president of Tsinghua University had repeatedly highlighted the pressing need for Tsinghua University to join this global trend. The top management’s understanding and vision were translated into a policy requiring Tsinghua University to maintain an institutional competitive advantage by urgently incorporating digital technologies into course delivery, content, and curriculum design. In 2013, the top university leadership team stated on multiple occasions that Tsinghua University, as a world-class university in China, should join the global MOOC movement (Document-201305). Although the concept of MOOCs originated outside China, university leaders coined the term "Chinese MOOC" to facilitate the launch of Tsinghua’s MOOC initiative in 2013.

#### Planning strategies and building infrastructure to combine explicit knowledge from in-and-outside the organisations

University leaders then hosted top-down, face-to-face meetings with selected faculty members to explain, communicate, and fine-tune the aims of the new approach to technology. Although some faculty members were concerned about the disruptive effects of online courses on their daily face-to-face teaching practices, others recognised their innovative potential. “I can intuitively smell the aroma of innovation,” recalling Prof. Heng, one of the pioneers of the MOOC initiative in this period.

Building on these discussions of the MOOC initiative, top management decided to build a MOOC infrastructure from scratch. The decisions made by top management were directly implemented by the middle and frontline staff, and in October 2013, China’s first MOOC platform, the XMOOC Platform, was launched (Document-201310). Faculty members from five different departments (two of them voluntary and the others so instructed) shifted their courses to the XMOOC Platform in 2013–2014. These courses included Financial Management, Ancient Chinese Architecture, Data Structure.

In summary, at this early stage, the MOOC was the major outside-in concept adopted by Tsinghua University. The top leaders were aware of it, introduced it to the institution, and prioritised its implementation. This supported a combination process with the promotion of MOOC initiatives and the creation of infrastructure (the Chinese MOOC platform) for the initiative at the organisational level (Graham et al., 2013; Nonaka et al., 2001). However, only a few stakeholders (e.g., designated faculty members and online project directors) noted and internalised the knowledge of the new technology through reading, listening, or indirectly observing others’ experiences during this stage. Some operational knowledge was developed, but it was limited in scale. Thus, the pace of change was very slow.

### Stage 2: Bottom-up early exploration through internalisation and socialisation (2014–2016)

#### Tinkering with MOOC teaching and BIP to embody explicit organisational knowledge in tacit knowledge on the individual level

From 2014 to 2015, except for the strategy of digital teaching initiative, the university seemed to be guided by the bottom-up, open-to-change, voluntary, and entrepreneurial principles. At this stage, some avant-garde faculty members tinkered with online teaching technologies in this stage. Prof. Heng, for example, explained that he fell into this experiment with his first open online course in 2014, but became a passionate advocate after his course proved to be one of the most popular courses on the XMOOC Platform. Prof. Heng continued experimenting with teaching strategies on the MOOC platform: “I invented smart gestures and body languages to improve the interactive experiences in the video-based teaching.” Another teacher, Prof. Xu, who became a leading expert in the later stages, reported that he offered his first MOOC on the XMOOC Platform, which inspired him to think about how to integrate his online courses with face-to-face instruction. In his opinion, flipped or blended learning with open courses offered a promising avenue for T&L innovation at Tsinghua University.

#### Sharing experiences of and passion for MOOC initiatives among frontline faculty

The tinkerers acted as seed teachers and gradually gained attention throughout the university. Some passionate teachers began asking about their tinkering practices, and people began to exchange experiences in coffee rooms (interviews with Prof. Fan and Prof. Yu). At this stage, knowledge sharing happened spontaneously, sometimes without the participants realising that they were actually sharing knowledge (Nonaka et al., 2006). This process also occurred when faculty members witnessed their colleagues designing MOOCs. For example, some participants mentioned that they had learned from colleagues how to use dialogical teaching strategies to design and deliver online courses (interviews with Prof. Geng and ST-Sang). In addition, the seed teachers’ passion and commitment to creating something new inspired some of their colleagues to experiment with the online education initiative in their daily practices (interviews with Profs. Yu and Fan). Thus, the socialisation process was found in informal meetings, collaborations, and pedagogical seminars.

In summary, individual faculty members functioned as intra-university entrepreneurs at this stage. In this bottom-up innovative approach, organisational knowledge was created by frontline faculty members who were exploring MOOC teaching and tinkering blended learning with their online courses. The internalisation and socialisation processes occurred when individual faculty members shared their experiences, both consciously and unconsciously, with colleagues (Graham et al., 2013; Nonaka et al., 2001). The knowledge gained by individuals tended to have personal features and was not easy transferred between teachers; however, the value of their openness towards each other, such as team-building effects, should not be underestimated (Geeraerts et al., 2016). Nevertheless, as these internalisation and socialisation processes did not occur for all faculty members at the university, the accumulation of localised, experiential knowledge to overcome challenges in online education was very partial.

### Stage 3: A Growing Middle-Up-Down Approach for Continuous Adoption and Innovation Through Externalisation (2016–2019)

#### Sustaining the vision by officially releasing digital learning action plans

A series of national policies for promoting online education innovation in Chinese higher education had been released since the 2015 academic year (Document-201504, 201606, 201707, 201806, and 201911). Although Tsinghua’s digital strategy vision has launched in January 2014 to revolutionise the pedagogical paradigm of the university through the large-scale adoption of MOOCs and BIP on campus (Document-201401), those national policies motivated the university to accelerate its small-scale experimentation with technology-supported education initiatives. This vision was supported by several digital learning actions focused on building institutional capacity for technology-based T&L. The university set up research grants (funding) for teaching innovations, recognised the extra teaching workloads for faculty engaged in MOOC- and BIP-related T&L innovations, adjusted the teaching evaluation scheme, and created extra technology and instructional support positions (i.e., student tutor positions). These measures provided strong official support and offered incentives to teachers who chose to jump on the technology bandwagon.

#### Facilitating the conversion of tacit knowledge into explicit concepts at the group level

Despite being formally founded in September 2013 under the Provost, the university’s Digital Learning Office guided and managed the initiative at this stage. This office had mixed responsibilities in relation to digital T&L practices within the institution, including proposing digitalisation plans for top managers, implementing the university’s digital visions, building and operating online resources, supporting faculty members and technical staff engaged in technology-supported instruction, and promoting digital learning research and domestic and international corporation in and across universities.

In addition to nurturing spontaneous networks among frontline faculty members engaged in MOOCs, the Provost and Digital Learning Office encouraged BIP and MOOC-based BIP explorations as a sustainable approach to TETL innovation. As part of middle management, they proactively organised lunch seminars and forums for faculty and staff to discuss common questions about BIP, including how to design and deliver BIP by integrating online courses (i.e., MOOCs) with face-to-face classroom instructions; how to build student learning communities in both offline and online learning environments; and how to evaluate student learning outcomes. Teachers, educational experts, and course chairs were invited to participate in seminars and forums. The externalisation process that transformed individuals’ tacit knowledge into explicit concepts occurred within these dialogical spaces. Faculty members (interviews with Profs. Xu, Geng, Yu, and Fan) confirmed that they discussed pedagogical skills, negotiated interpretations of different forms of instructions, and revised their BIP lesson plans continuously during this period.

#### Verbalising tacit knowledge to expand knowledge externalisation

In addition to face-to-face interactions in seminars and forums, externalisation also occurred through the creation of digital platforms for sharing good practices. The Provost and Digital Learning Office recognised the value of having faculty and staff share good practices. They then collected and systematically documented innovative teaching practices through digital platforms (i.e., digital newsletters or WeChat public accounts). Documents on these platforms, including first-hand accounts of champion teachers’ expertise, could be accessed, learned from, and adopted by a wide range of colleagues (Lieberman & Pointer Mace, 2009). As the Digital Learning Office pointed out, documenting successful cases would not only benefit the office and faculty members with personal experience but also motivate more people to join the movement (interview with A-Lan).

#### Setting up a task-force to experiment, synthesise, and process explicit knowledge into new explicit knowledge

To further integrate technologies into the entire learning trajectories of students and support BIP by enriching teacher–student interactions, the Digital Learning Office assembled experts in different fields (i.e., educational technologists, faculty from various disciplines, and instructional designers) to set up a university-level task force for the development of a new interactive LA tool. They first discussed the need for a new instructional technique that would reveal students’ learning behaviour to teachers and instructional designers, provide just-in-time support and feedback for students, motivate interactions (Herodotou et al., 2020), and more importantly, be accessible to all faculty members and students. This creative and essential dialogue about a desired new tool was an important part of the knowledge externalisation process.

To reach the target, the task force eventually decided to create a plug-in for PowerPoint files in tablets and an external link on WeChat (a popular communication app for mobile phones). This open access design provided both teachers and students with straightforward access to the tools, and closely connected them during and after classes (interviews with Profs. Xu and Fan, ST-Lee, ST-Hu, ST-Tu, ET-Gang, and ET-Xue).

When the prototype of RClass launched in 2016, it underwent a comprehensive, back-and-forth bug-testing and updating process. The university used its technical infrastructure and technical expertise to integrate it into the BIP over a 3-4-year period. Bug-testing was a multifaceted and iterative activity involving learning designers, IT workers, and students collaborating with each other to assist the primary users (i.e., the teachers) in learning and exploring the tools (interviews with Prof. Xu, ET-Gang, and ET-Xue). Documents and databases on this LA tool and its services have gradually been accumulated (Document-201812, 201912). During 2018–2019, the evolution of this LA tool, which supported classroom instruction in university contexts, emerged as a combined process.

In summary, at this stage, the university’s digital vision was focused on accelerating experimentation with technology-supported education and making technology a catalyst for transforming the pedagogical paradigm of university. Whereas the university’s top and middle management took leadership roles, the visible knowledge actors were in middle management (Giddens, 1984; Nonaka et al., 2001). They translated abstract visions into more concrete concepts, facilitated interactions between tinkerers, and motivated more frontline faculty members to join the trend and share their experiences. These leadership strategies supported a strong externalisation process within the campus (Donate & de Pablo, 2015; Von Krogh et al., 2012). In the meantime, a task force with multi-dimensional stakeholders negotiated, experimented with, and explored a new LA system. The creation of the RClass system followed a fluctuating iterative trajectory. However, the number of adopters gradually increased between 2018 and 2019, indicating that the combination process occurred gradually as people increasingly used the tool and successively engaged in its iterative updating (Paavola et al., 2004).

We classified this stage as the growing middle-up-down approach for continuous adoption because the Provost and Digital Learning Office presented a gradual concerted effort to create and energise interactions among frontline faculty and multiple stakeholders and to stimulate knowledge sharing on both interpersonal and digital platforms. The accumulation of conceptual knowledge in this stage created a strong knowledge base for further innovation (Nonaka et al., 2006).

### Stage 4: A Strong Middle-Up-Down Approach for Mature Implementation & Innovation Through Expansive Combination and Internalisation (After early 2020)

#### Pandemic as an unfolding opportunity to assemble internal and external knowledge resources

In the early spring of 2020, China was the first country to announce a comprehensive national online education plan in response to the COVID-19 pandemic. Tsinghua University was the first Chinese university to release a new version of its contingency plan and move T&L entirely online.

In early February of 2020, the university president delivered an online lecture as part of a lively synchronous online course supported by the RClass system. Nearly 50,000 students, faculty, staff, and alumni flooded the virtual lecture hall and tested the system. In the lecture, top management encouraged faculty and students to continue learning during the pandemic and explore TETL. They indicated that Tsinghua University saw this unexpected and extreme digital shift as a valuable opportunity to support sustainable digital innovation throughout the whole institution. Since middle February 2020, 4,254 Tsinghua courses taught by 2,681 faculty members to more than 25,000 students have been delivered online (Document-202002), suggesting a remarkable internalisation process.

#### Expanding joint efforts to synthesise explicit knowledge into complex and systematic sets of explicit knowledge

During this period, knowledge combination processes occurred in the cross-functional teams that developed various conceptual and systematic knowledge products. An Ad-hoc Online Teaching Committee including administrators, coordinating directors, program directors, instructional experts, and champion teachers was established by the Provost and Digital Learning Office over 12 days. The committee members synthesised existing knowledge and co-created a hybrid model of online education that integrated synchronous and asynchronous online and offline courses by conceptualising technology-supported T&L in the context of an urgent transition to online teaching. Several online teaching blueprints, manuals, handbooks, Q&A systems, and short demo videos on hybrid instructional practices were produced over 3 weeks as learning resources for faculty and students (interviews with Prof. Xu, A-Lan and A-Nong).

The task force for RClass development was enlarged and an R&D group with a multidisciplinary background became involved. They conducted interdisciplinary research projects during the pandemic, including evidence-based evaluations of online education, the development and application of e-assessment tools, and consultations with internal and external stakeholders to inform future policies (interviews with A-Lan and A-Nong).

In addition, a second task force, called the Online Teaching Assistant Team, was assembled in 2–3 weeks. More than 100 technical volunteers and 400 volunteer student tutors were recruited and trained to provide timely and comprehensive technical and pedagogical support to the faculty and students, such as organising online discussions, answering questions and solving technical problems. These volunteers, who were mainly students with excellent digital literacy, acted as powerful agents to ensure the quality of the online courses (interviews with ST-Lee and ST-Hu). They served as a bridge between policy implementation and frontline teaching practices, according to the Director of the Digital Learning Office (interviews with A-Lan).

#### Scaling up knowledge products to disseminate in and across organisation boundaries

The combination process expanded when Tsinghua University implemented online teaching modules through integrating with the RClass system. As the CEO from the XMOOC Platform and the RClass system stated, “during the early pandemic period, the flow of the RClass system increased 17 times because of the upscaling of tools” (Interviewee ET-Gang, A-Lan). Information and communication technologies also facilitated the process of combination (Geeraerts et al., 2016). We observed that technology developers working extensively with teachers to use real-time student data to inform their T&L practices.

Moreover, these knowledge products rooted in the Tsinghua University campus served as boundary objects (Akkerman & Bakker, 2011) that could be disseminated to other universities nationwide. For example, the hybrid online teaching module co-created by the Online Teaching Committee was upgraded to the ‘Twin Class’ module, which could be used synchronously by other domestic universities. Over 2,800 students from other colleges were recorded as enrolling in more than 30 Twin Classes in the 2020–2021 academic year. Many participants reported that the scaled-up classes enriched their learning experiences, although there are still many infrastructural limitations in some parts of China (Document-202003, 202007), which echoes other countries’ experiences that the lack of a well-developed ICT system may explain the invisible improvement of local ICT education and industry (Mugruza-Vassallo & Suárez, 2016).

In summary, Tsinghua University was the first Chinese university to have been well prepare to move T&L entirely online. It also served as a national driver of educational innovation by turning the crisis into an opportunity in response to COVID-19. As noted, the time-consuming and fluctuating process of knowledge externalisation (Stage 3) allowed Tsinghua University to accumulate the rich knowledge base and human resources needed to seize the opportunity for a rapid expansive combination in response to the COVID-19 pandemic. In addition, knowledge products rooted in the Tsinghua campus served as boundary objects for disseminating to other national HEIs to leverage their change and innovation. Some points of view could be highlighted regarding this stage. First, top leaders and administrators clearly re-defined the digital vision and facilitated the expansive knowledge combination and internalisation of technology innovations throughout the entire institution and even beyond (Nonaka et al., 2001). Second, we observed a strong middle-up-down approach of the staff of the Provost and Digital Learning Office in coordinating various functional teams and practicing knowledge management in this stage: they built intimate collaboration with the top to create plans and facilitated accelerated knowledge combination (i.e., blueprints and manuals) and knowledge creation (i.e., Twin Classes) in and across Tsinghua University. They developed their ability to collaborate with stakeholders horizontally and vertically, to create new concepts (i.e., hybrid online education models, cross-functional teams, Twin Classes) to bridge the gaps between theory and practice, and to facilitate the expansive technology innovation in university contexts (Nonaka et al., 2006; Von Krogh et al., 2012; Zhang et al., 2020) They then played an important role in synthesising the externalised concepts as new knowledge products (i.e., Twin Classes) and disseminating them across university boundaries (Akkerman & Bakker, 2011; Paavola et al., 2004).

However, the university still faces challenges in continuously improving the quality of technology-supported T&L and in routinising the innovations embedded in the institution (Rogers, 2003; Yang & Huang, 2021). These challenges may require the university to undergo another cycle of knowledge creation to innovate from the inside out.

## Discussion

In this study, we combined the four modes of knowledge conversion with the diffusion of the TETL innovation framework to identify the four stages of technology innovation in one university, which occurred over seven years (shown in Table 4). The main contributions of this study are as follows.

First, our study extends on the institutional innovation research by identifying that the uptake and diffusion of TETL at Tsinghua University represent an organisational knowledge creation process. Previous literature examined technology adoption and innovation with a focus on individual faculty members (especially tinkerers and champions who have been pioneers or central to this journey) (Ashrafzadeh & Sayadian, 2015; Fraser, 2019; Kopcha et al., 2016); or on institutions and concluding that universities can accumulate multifaceted resources (e.g., infrastructure, availability of data, institutional culture, buy-in from stakeholders) (Herodotou et al., 2020; Porter et al., 2016); or on developing both people (institutional capacity-building) and knowledge products or artifacts (online technologies, materials or courses creating) in terms of TETL adoption (Aitchison et al., 2020; Vigentini et al., 2020). We observed that top management awareness promoted TETL initiatives and the creation of an infrastructure (XMOOC Platform) based on external concepts that emerged from a quick combination process (stage 1). Limited internalisation means that few new ideas were generated from the localised context during this stage. Early exploration (stage 2) was driven by a few leading-edge faculty and their unintentional bottom-up entrepreneurial teaching innovations. These internalisation and socialisation processes helped people create more experiential tacit knowledge about TETL innovation; however, they failed to continuously develop into collective knowledge shared by large-scale faculty members until strong externalisation happened (stage 3), whereupon they moved to the expansive combination (stage 4). Apparently, the diffusion of TETL innovation in Tsinghua University from tinkerers to institutional innovation evolved as the knowledge created by individuals (tinkerers and champions) and triggered by technologies (online course platforms and learning technologies) became available and was amplified, diffused, connected, and embedded into the organisation’s knowledge system (Brix, 2017; Nonaka et al., 2006; Rogers, 2003).

Second, this study nails the critical impact of collaborations and the generative power of technological tools and products, which leverage TETL innovation through knowledge externalisation toward organisational level. From 2016 to 2019, we observed a time-consuming and fluctuating process of knowledge externalisation that allowed Tsinghua University to accumulate a rich knowledge base and the human resources required to seize the opportunity for a rapid expansive combination in response to the COVID-19 pandemic. This finding demonstrates that on the one hand the externalisation process which hinges on different stakeholders with a mix of specific knowledge and capabilities collaborating and sharing knowledge (Nonaka et al., 2006), accumulates rich conceptual knowledge (through tacit to explicit). On the other hand, information technology infrastructure and digital learning tools have also evolved to accelerate the knowledge externalisation process (e.g. sharing good practices through the creation of digital platforms) by reducing well-known obstacles (such as using an economic method of scaling across universities) to disseminate conceptual knowledge as collective knowledge (the new knowledge developed in interaction with others may have exceeded that of any individual’s personal knowledge) (Braßler, 2020; Grossman et al., 2001) in and across universities. Both collaborations and technological objects facilitating a rich externalisation helps to stimulate knowledge combination processes, in which ‘distributed cognition’ (Grossman et al., 2001, p. 975) is formed. This distributed cognition seemed to enable faculty members and stakeholders to identify and co-create novel ways of knowing in terms of TETL innovation (Vigentini et al., 2020) that renewed the systematic knowledge of the university.

Third, this study reveals the unique role of middle leadership in TETL innovation. This extends the diffusion of the TETL innovation framework (Graham et al., 2013; Rogers, 2003) to uncover the agency of middle management in motivating stakeholders to become involved in the co-creation of technology initiatives (Tsai et al., 2021; Vigentini et al., 2020). Our study demonstrated that middle leaders (consisting of faculty members who served as online teaching committee members, technological professionals, department chairs, educational researchers, and administrators) facilitate collaboration between the top (university leadership), middle (committees, task forces), and practitioner (faculties, staffs and students) levels. They can identify the challenges and opportunities TETL initiatives within their universities, create appropriate concepts (e.g., hybrid online education models, cross-functional teams) to address the gaps between structural properties and social practices; strategically promote interactions between diverse social groups; and disseminate knowledge products in and across organisations. They were observed to manage both explicit knowledge through exchange and combination, and tacit knowledge through communication and use in promoting teaching innovative contexts within HEIs (Donate & de Pablo, 2015; Zhang et al., 2020). We found that middle leaders in Tsinghua University are knowledgeable actors (Giddens, 1984; Schophuizen et al., 2018). They can practice knowledge-oriented leadership to deliberately guide knowledge workers to learn and use knowledge (Ribiere & Sitar, 2003) while sensing and seizing opportunities to innovate (Teece, 2009). These leadership strategies help promote transitions between early and continuous adoption (stage 2, 3) and mature innovation for institutional innovation (stage 4).

## Conclusion and implications

**Fig. 1:**
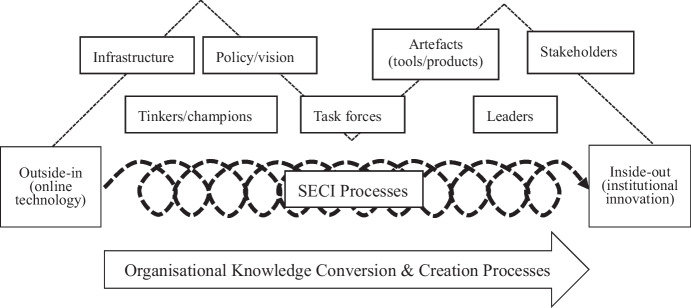
Technology-enhanced teaching & learning innovation as organisational knowledge conversion & creation processes

This study foregrounds TETL innovation as an organisational learning and knowledge creation process underlying the visible factors of people and products (Fig. 1). It demonstrates how the diffusion of TETL innovation in the University under investigation from the efforts of tinkerers to institutional innovation evolves as the knowledge created by individuals (tinkerers and champions) and triggered by technologies (online course platform and learning technologies) becomes available and is then amplified, diffused, and embedded into the organisation’s knowledge system, which makes institutional change happen. Technology, as an outside-in factor, may leverage people within HEIs to learn, interact, and overcome the challenges in fulfilling digital T&L innovations from the inside out. However, we do not claim that this is an universal model that guides all T&L innovations at universities. This approach can serve as a heuristic framework for other cases or alternative models.

The following implications are offered for optimising and leveraging the diffusion of TETL innovation in HEIs, which might interest university leaders as well as researchers in the future.

First, university leaders should be clear on the purpose for innovation. In our study, successful implementers began with an administrative advocate who convinced others of the value of pursuing TETL innovations. Moreover, successively redefining and modifying the goal of innovation facilitates resource provision, prevents barriers related to institutional policies, and develops appropriate governance structures that support the scaling-up and diffusion of innovation.

Second, university leaders should consider and strategically manage their knowledge when seeking for whole-institutional and sustainable innovation. The diffusion of TETL innovation in HEIs is not merely the development of people (institutional capacity-building) or knowledge products and artifacts (making online technologies, materials or courses), but also a knowledge management initiative that seeks to co-create innovation. It relies on interactions between faculty members who can expand the boundaries of individual knowledge, people from different areas of the university with diverse social practices for knowledge externalisation, and human agents and technological objects that may form a sociotechnical system within the university.

Third, we emphasized the middle-up-down leadership approach in term of sustainably facilitating certain types of collaboration and strategically practicing their knowledge-oriented leadership strategies to leverage innovation. Future studies could examine how to build middle management capacities and leadership practices for knowledge creation, specifically to initiate, disseminate, and sustain TETL innovations in higher education.

## Data Availability

The data that support the findings of this study were collected by the authors, and are available from the authors but restrictions apply to the availability of these data, which were used under ethical approval of the informants for the current study, and so are not publicly available. Data are however available from the authors upon reasonable request and with permission of the informants.

## References

[CR1] Aitchison (2020). Tensions for educational developers in the digital university: Developing the person, developing the product. Higher Education Research & Development.

[CR2] Akkerman S, Bakker A (2011). Boundary crossing and boundary objects. Review of Educational Research.

[CR3] Ashrafzadeh A, Sayadian S (2015). University instructors’ concerns and perceptions of technology integration. Computers in Human Behavior.

[CR4] Bøe T (2021). Continued use of e-learning technology in higher education: A managerial perspective. Studies in Higher Education.

[CR5] Braßler M (2020). The role of interdisciplinarity in bringing PBL to traditional universities: Opportunities and challenges on the organizational, team and individual level. Interdisciplinary Journal of Problem-Based Learning.

[CR6] Brix J (2017). Exploring knowledge creation processes as a source of organizational learning: A longitudinal case study of a public innovation project. Scandinavian Journal of Management.

[CR7] Coates H (2022). A turning point for Chinese higher education.

[CR8] Donate, de Pablo (2015). The role of knowledge-oriented leadership in knowledge management practices and innovation. Journal of Business Research.

[CR9] Fraser S (2019). Understanding innovative teaching practice in higher education: A framework for reflection. Higher Education Research & Development.

[CR10] Frei-Landau R, Muchnik-Rozanov Y, Avidov-Ungar O (2022). Using Rogers' diffusion of innovation theory to conceptualize the mobile-learning adoption process in teacher education in the Covid-19 era. Education and Information Technology.

[CR11] Geeraerts (2016). Teachers' perceptions of intergenerational knowledge flows. Teaching and Teacher Education.

[CR12] Giddens A (1984). The constitution of society: An outline of the theory of structuration.

[CR13] Graham (2013). A framework for institutional adoption and implementation of blended learning in higher education. The Internet and Higher Education.

[CR14] Grossman (2001). Toward a theory of teacher community. Teachers College Record.

[CR15] Herodotou (2020). The scalable implementation of predictive learning analytics at a distance learning university: Insights from a longitudinal case study. The Internet and Higher Education.

[CR16] Kopcha (2016). Understanding university faculty perceptions about innovation in teaching and technology. British Journal of Educational Technology.

[CR17] Krogh V (2012). Leadership in organizational knowledge creation: A review and framework. Journal of Management Studies.

[CR18] Labaree R (2002). The risk of ‘going observationalist’: Negotiating the hidden dilemmas of being an insider participant observer. Qualitative Research.

[CR19] Lieberman A, Pointer Mace D (2009). Making practice public: Teacher learning in the 21st century. Journal of Teacher Education.

[CR20] Liu (2020). Understanding academics' adoption of learning technologies: A systematic review. Computers & Education.

[CR21] Louvel S (2013). Understanding change in higher education as bricolage: How academics engage in curriculum change. Higher Education.

[CR22] Mugruza-Vassallo, C. A., & Suárez, S. M. (2016). Academia and patents at information and communications technology in South-America productivity. *2016 6th International Conference on Information Communication and Management (ICICM), IEEE*, Hatfield, UK, 2016, 24–29. 10.1109/INFOCOMAN.2016.7784209

[CR23] Nonaka, Dierkes M, Antal AB, Child J, Nonaka I (2001). A theory of organizational knowledge creation: Understanding the dynamic process of creating knowledge. Handbook of organizational learning and knowledge.

[CR24] Nonaka (2006). Organizational knowledge creation theory: Evolutionary paths and future advances. Organization Studies.

[CR25] Ocak MA (2011). Why are faculty members not teaching blended courses? Insights from faculty members. Computers & Education.

[CR26] Paavola (2004). Models of innovative knowledge communities and three metaphors of learning. Review of Educational Research.

[CR27] Pinho C, Franco M, Mendes L (2021). Application of innovation diffusion theory to the E-learning process: Higher education context. Education and Information Technology.

[CR28] Polanyi M (1966). The tacit dimension.

[CR29] Porter (2016). A qualitative analysis of institutional drivers and barriers to blended learning adoption in higher education. The Internet and Higher Education.

[CR30] Ribiere, Sitar (2003). Critical role of leadership in nurturing a knowledge-supporting culture. Knowledge Management Research & Practice.

[CR31] Rogers E (2003). Diffusion of innovations.

[CR32] Salmon, G. (2005). Flying not flapping: A strategic framework for e-learning and pedagogical innovation in higher education institutions. *ALT-J 13*(3), 201–218. 10.1080/09687760500376439

[CR33] Schophuizen (2018). Eliciting the challenges and opportunities organizations face when delivering open online education: A group-concept mapping study. The Internet and Higher Education.

[CR34] Songkram N, Chootongchai S (2020). Effects of pedagogy and information technology utilization on innovation creation by SECI model. Education and Information Technology.

[CR35] Teece D (2009). Dynamic capabilities and strategic management.

[CR36] Tsai (2019). Complexity leadership in learning analytics: Drivers, challenges and opportunities. British Journal of Educational Technology.

[CR37] Tsai (2021). Connecting the dots: An exploratory study on learning analytics adoption factors, experience, and priorities. The Internet and Higher Education.

[CR38] Vigentini (2020). Evaluating the scaling of a la tool through the lens of the sheila framework: A comparison of two cases from tinkerers to institutional adoption. The Internet and Higher Education.

[CR39] Watermeyer (2021). Covid-19 and digital disruption in UK universities: Afflictions and affordances of emergency online migration. Higher Education.

[CR40] Yamagata-Lynch (2015). Transforming disruptive technology into sustainable technology: Understanding the front-end design of an online program at a brick-and-mortar university. The Internet and Higher Education.

[CR41] Yang, B., & Huang, C. (2021). Turn crisis into opportunity in response to covid-19: Experience from a Chinese university and future prospects. *Studies in Higher Education,**46*(1), 121–132. 10.1080/03075079.2020.1859687

[CR42] Zhang X (2020). The new historical divide of online education: Dialogues with key leaders during the epidemic. ECNU Review of Education.

